# Report on the Homœopathic Treatment of Acute Diseases in Dr. Fleischmann's Hospital, during the Months of May, June, and July, 1846

**Published:** 1846-10

**Authors:** George W. Balfour


### III. REPORT ON THE HOMŒOPATHIC TREATMENT OF ACUTE DISEASES IN DR. FLEISCHMANN'S HOSPITAL, VIENNA, DURING THE MONTHS OF MAY, JUNE, AND JULY, 1846.

BY GEORGE W. BALFOUR, M.D. EDIN.

(*In a Letter to Dr. Forbes.*)

Vienna, August 14th, 1846.

My Dear Sir,—I shall now proceed to lay before you the results of my inquiry into the practice of homœopathy, prefacing them with a short account of its present state in Germany, where it is now become quite fashionable, and nowhere more so than in Austria. Even travelling physicians are now chiefly chosen from among its followers, who are, consequently, far from being insignificant in numbers. No young physician settling in Austria, excluding government officers, can hope to make his bread, unless at least prepared to treat homœopathically, if requested; and many, after attempting to do so, return to Vienna to make themselves acquainted with this new method. Many older men also attempt, by thus conforming to the foible of the day, to recruit a failing practice. Thus homœopathy is studied, not for any beauty or truth to be found in its doctrines, but from necessity, for a livelihood. Many continue to practise both methods, not eclectically, but according to the wish of the patient, believing in neither, leaving inquiry to others, and stumbling blindly on. Others, confident in homœopathy, merely use ordinary medicine, or, as it is termed, allopathy, in so far as occasionally to give a laxative, or, where the relatives urge it, to bleed—all by way of *placebo;* and I believe there are few, except the older and better established practitioners, who would not give such a *placebo* if requested. Nay, the inclosed recipe will show that even they are not altogether free from blame in this respect, or else that they have found their guiding principle in many cases false. The gentleman who gave me it (an allopathic physician) told me he might have procured many such.

While thus, from force of circumstances, everywhere increasing their domains, homœopathists are far from sitting idly down, content in following the footsteps of their first great master. Imbued with the progressive spirit of the age, they also strive after improvement, and while professing to retain "*similia similibus*" as their fundamental principle, are endeavouring to advance their method, and give it a more permanent and dogmatic character. Seeing, as it would seem, that the above-mentioned principle does not suffice for every case, they change the name of homœopathy to *Specific Medicine.* These men are dissatisfied with Hahnemann's work on the Materia Medica, on account of the imperfect nature of the observations, and look upon it as an enormous and almost unreadable catalogue of symptoms, more fitted for the memory than the intellect, and thus not only rendering the practice of homœopathy more difficult for his own followers, but throwing an almost insurmountable obstacle in the way of physicians who think otherwise, and often preventing those very intellects best fitted to become leaders in the reformed practice from ever studying it. (Œsterreichische Zeitschrift für Homöopathie, 1 Band, 1 hft. s. 4-5). Accordingly they have commenced a careful re-proving of all the medicines, with the view of obtaining, not a mere catalogue of symptoms, but a collection of medicinal diseases. A journal (the above-quoted) is also published in Vienna, with the view of giving publicity to these provings. This shows them to be in earnest in their endeavours to simplify their method and render it more practicable, as any one will be more inclined to confess, after having looked into Jahr's Codex, the best of the day. Who can wonder at the difficulty experienced by homœopathists in choosing their remedies, when we find the same symptoms repeated, it may be, in a somewhat different order, under the head of almost each separate medicine? Nay, Isensee (Geschichte der Medecin, vol. vi, p. 1569) goes so far as to say, that in no case are the peculiar and characteristic operations of a medicine to be found, except in such cases as Hahnemann has, from want of original observation, borrowed from the allopaths; and that his own symptoms may all be referred to sobriety, fasting, ill-humour, and sleepiness, caused by continued attention to—*nothing*, mixed with those innumerable sensations which crowd every hour of our life!

Among those who are guided by the same principle in their choice of a remedy, the dose in which this ought to be given has formed, and is forming, a fruitful source of internal contention. Many, amongst whom may be reckoned Fleischmann and most of the Vienna homœopathists, employ the modern formula of 10 : 90 in making their dilutions, and seldom go higher than the 3d or 4th perhaps, but more rarely the 6th. Others, contending that those dilutions are too low and apt to produce disagreeable consequences, employ from the 14th to the 30th; while yet others, outstripping Hahnemann himself in their idea of the effect of friction and shaking in developing the latent powers of remedies, contend that these are at first produced too powerfully and irrestrainably, and, when employed in those low dilutions, only render the disease worse, without in the least tending to cure; but that by being further diluted (*potenziren* is the technical term) the agent at length arrives at that pitch at which, according to their ideas, the whole remedial power is developed, but so mild and tractable, that it at once cures without producing any exacerbation (verschlimmerung), and is therefore—and then alone—entitled to our confidence and the name of remedy. The champion of this sect is Dr. Grosse, a practising physician, at Jütterbock, whose usual dilutions are 2, 4, 8, and 900; and he often contents himself with allowing the patient to smell the remedy—whether one or more globules at one time I am not aware—waiting patiently for four weeks or so, for the completion of the cure, not even permitting a second smell: so mild, yet certain is the remedial action! This method, however, does not pass current with the homœopaths generally; it is rather too finely drawn for even their sensitive imaginations. A paper by Dr. Grosse, urging the importance of this mode, and accompanied with cases to prove its advantages, was published in the 21st volume of the 'Homœopathic Archives,' and has been criticised in a late number of the 'Austrian Homœopathic Journal,' by Dr. Böhm, a practising homœopathic physician in Vienna, and his cases in proof set aside on the grounds—1st, that in those cases in which a cure seems best made out, lower powers produce equally good results—hence an immense saving of time to the physician; 2d, that many cases recorded as cures seem to be merely a longer than usual intermission between paroxysms, which may, nay, often does occur, without any previous treatment; 3d, that many others were merely the natural result of the lapse of time—e. g. a severe pain in the foot which took its departure four weeks after smelling phosphor. 200; 4th, in others the diagnosis seems to have been incorrect, and the credit due to Nature given to the remedy. Dr. Fleischmann, also, in a note says, with respect to this mode, "Are the results favorable I grieve that truth appears so decked in folly's garb, as to drive many from her in disgust. Are they imaginary? I grieve again, that spectres arise before which the soberest must retire in fright."*

This Dr. Grosse seems but the pioneer for newer and bolder discoveries in this vast and unknown field; for another writer speaks of arsenic ·2000.

* Might not all this be with equal truth applied to homœopathy generally? It is to be wished that common homœopathists would as carefully criticise their own cases, and cease to imagine that "quia post, non propter" refers only to allopathic and high-power cases.—G. W. B.

What would Dr. William Wood say to this, if 30 proved too much for his weak faith and still feebler imagination? At this rate the dilutions of one remedy alone would, with the requisite utensils, form no inconsiderable laboratory, and these preparations consume no trifling portion of valuable time, not to speak of the innumerable trials necessary to be made, in order to find out in which dilution, from ·1 to ·2000, the remedial powers of the agent are most fully and usefully developed; verily, *ars longa, vita brevis.*

There is still another sect, or at least an individual, in Vienna, Dr. Georg Schmid, who has within the last few months given to the world a volume, as the result of many years' experience in homœopathic practice, with respect to the preparation of medicines and the amount of the dose (Ueber die Arzneibereitung und Gabengrösse, Wien, 1846). The latter portion of the work possesses peculiar interest, inasmuch as the author contends, from personal experience, that the present small doses are worse than useless, and that the mother tincture, 1 drop for a dose, or larger doses than usual of the 1st or 2d triturations (verreibungen) ought to be employed. These triturations he employs not because he considers that remedial power or new chemical properties—as solubility—are thereby imparted, but because the medicine being thus divided into its finest atoms, and each particle thus brought into immediate contact with the organism, is better enabled to exert its remedial agency. He adds (p. 60), that by using this means of division, we have not an absolute increase of power, but rather, with the diminished mass, a diminution of it. So long as the smallest trace of the remedy is perceptible, so long can we understand that such a dilution may be efficacious; but when the medicine is neither to be recognised by chemical nor physical means, and probably never will be, how is its efficacy to be explained? His remark upon homœopathic exacerbation deserves to be carefully read by every homœopath. He states that Hahnemann, in his preface to his work on Chronic Diseases, says that nothing worse could happen than that the small doses did not *help*, for *hurt* they could not. "Is it not," asks Schmid, "the duty of a physician to be of positive use to his patient, that is, to help? To do this he must use the proper remedies in the proper doses. But he can also injure; therefore medicine is no child's play, but the life-task of the physician; and he who feels not the power and the courage to discharge this duty, ought to choose some other profession. As there is no means of distinguishing the medicinal disease from the real, so the physician may ascribe an exacerbation which arose after taking his physic, to this cause, though it may be totally unconnected with it; and by then diminishing the dose, may allow this exacerbation to proceed unchecked. How often has not this already happened! Indeed it would not be difficult to discover amongst the many published cases of so-called medicinal exacerbation, merely the progressive increase of the disease, over which the medicine had not the slightest influence, being either of the wrong substance or deficient in quantity; yet the physician has reduced the dose, giving over his patient to nature, it may be to death." (pp. 227, &c.)

Dr. Schmid even turns Hahnemann against himself, showing that of the only four of his cases which have been published, two having been treated with the mother tincture, and two with various dilutions, the first two alone are entitled to the name of cures. Again, Hahnemann, after quoting allopathic cases in favour of his principle, adds, "large doses, though dangerous, often cure without peculiar disadvantages." (Organon 4 Aufl.s.54-104.) Finally, Rau—though his work be no longer new—may be quoted as constituting one step towards the formation of an eclectic school; and doubtless, his remarks have afforded much comfort to those homœopaths who have been led astray by interest. He acknowledges that there is much good in the old school, nay, that "an antipathical practice may indeed be thought of." (Organon der Specificischen

Heilkunst, s. 45, 46.) He goes on to say that venesection, derivative and repulsive remedies, may all at times be useful; and quotes cases from his own practice, illustrating the fact that symptoms not to be relieved by homœopathic remedies will often yield at once to purges or emetics.

The Homœopathic Hospital of Vienna is a private one, in the convent of the Sisters of Charity: it was erected by them, in the first instance, with the view of thereby obtaining practical instruction in what constitutes their chief object—the cure of the sick. They have also another smaller hospital in a sister convent in the suburb Leopoldstadt. The homœopathic hospital is situated south-west of the city, in the suburb Gumpendorf, near the lines, much farther from the town than the General Hospital, and upon a loftier and airier situation; the latter being situated on the banks of the brook Alser, which has, within the last year or two, been covered over. The homœopathic hospital is also less than the other, containing only 50 beds—25 for males, and 25 for females, divided amongst four well-lighted and well-aired rooms. A few cribs for children are also occasionally occupied.

From its opening in July 1832, till July 1833, Dr. Mayerhoffer was its medical attendant. During the early part of this period the cholera was ravaging Vienna; and during the prevalence of this disease Dr. Mayerhoffer established his confidence in homœopathy. After him came the above-mentioned Dr. Schmid, who treated the patients, indeed, according to the homœopathic principle, but not with the wonted small doses. He continued till January 1835; at which time Dr. Fleischmann commenced those duties which he still continues to discharge, and whose name, in connexion with homœopathy and this hospital, is now known over both the old and the new worlds; and from whom the practice of homœopathy has received a greater impulse than from any other since the days of Hahnemann. During the first appearance of cholera here, the practice of homœopathy was first introduced; and cholera, when it came again, renewed the favorable impulse previously given,—as it was through Dr. Fleischmann's successful treatment of this disease that the restrictive laws were removed, and homœopathists obtained leave to practise and dispense medicines in Austria. Since that time their number has increased more than threefold in Vienna and its provinces.

The medicines employed in this hospital are all prepared in it by lady-apothecaries (apothekerinnen), who, for this purpose, receive a special education, and undergo an examination. None of the drugs are obtained from allopathic apothecaries; or, if so, are carefully tested and purified. The tinctures are either made in the hospital, or obtained from those homœopathic physicians who reside where the plants are indigenous. The text-book for the preparation of the tinctures and dilutions is Gruner's 'Homœopathic Pharmacopœia.' Gruner employs the proportions of 10 to 90 in making the latter, or 5 to 95 in the case of such salts, oils, &c., as are not soluble in the above proportion. This dilution is not marked 1, but fractionally, and a double portion 20 : 80, taken to form the second dilution, so as to bring it again into the proper relative proportion. Hahnemann's empirical rules as to rubbings, scrapings, and shakings are discarded. The time employed for the first two is regulated by the relative hardness and adhesiveness of the material; and in making the dilutions the shakings are continued only till the whole is clearly dissolved; when the dilutions are commenced by trituration with sugar of milk, 3 such are made, and one part of the third added to 9 of distilled water to make the fourth. The fifth dilution is made by the addition of one part of the fourth to nine of watered spirit; i. e. spirit containing 70 per cent. alcohol, mixed with an equal quantity of water; and the further dilutions are continued with this spirit. The temperature recommended is 12° to 15° R. The medicines are administered to the patients either in powder or solution, according to the frequency with which they are to be given. For the first method, a drop of the solution, or a grain of the trituration directed, is mixed with a small quantity of sugar of milk for each dose; for the latter, a small bottle-ful of water is taken, and the trituration dissolved, or the solution dropped into it in the proportion of 2 grains or 2 drops to each ounce, half an ounce being the dose. Those for whom no medicine is considered requisite, get a powder of sugar of milk, or something of that kind. Where two medicines are ordered, they are given alternately at intervals of two or three hours. The diet is light and simple; no coffee, tea, or wine is allowed; the latter, however, is sometimes given in cases of old people, or in convalescence from severe disease, if thought requisite. In acute diseases a light soup is given three times a day, exchanged, on commencing recovery, for a more nourishing one (eingekochte suppe), and the diet so progressively improved. No food is allowed to be given during treatment in which acid is predominant.

In taking into consideration the adjuvants to treatment, the religious character of the establishment must not be forgotten. The greater part of the patients are Roman Catholics. These find themselves surrounded by all the consolations of religion—by everything which, in their opinion, tends to ensure, in the event of death, a speedy passage of the soul to the realms of bliss. Their minds, thus set at ease with respect to futurity, are less gloomy and desponding, and consequently react less unfavorably upon the body than in the opposite circumstances. Nay, looking upon their nurses as self-devoted in the service of heaven and of suffering humanity, they cannot but believe that the blessing of the Almighty will rest upon their labours; and being the object of those labours, they naturally enough appropriate a portion of this blessing to themselves, and imagine that their recovery can hardly fail to be promoted by their being the inmates of such an institution. In support of this opinion, I may state that although most of the patients were young, and many dangerously ill, I never heard one expression indicative of a fear of death, nor one murmur, however slight, unless extorted by the extremest pain, and even then it was more an aspiration after relief than a grieving at suffering. The severer the disease, the more closely do they grasp their rosaries and crucifixes. So long as they are able to read, prayer-books are constantly in their hands, and even in the intervals of delirium, nay, often, in the cases of women especially, during delirium itself, the exercise of repeating prayers, or snatches of them, is their occupation. The superiority of attendance is also one great advantage in favour of this hospital, independently of the important fact just stated, that the nurses are spiritual as well as temporal comforters. It may not be inappropriate to state here also—more particularly as I shall have occasion to compare the results of the homœopathic treatment with those observed in an ordinary hospital—that in Dr. Fleischmann's institution the pneumonic patients are *not* ausculted and percussed, and unceremoniously lectured over several times a day, as is the case in the General Hospital. This difference will not appear a matter of indifference to any one who has witnessed the proceedings in the two instances.

The comparative youth of the patients in this hospital must also be taken into consideration. This is at a glance evident to the visitor, and will be rendered so to you, when I state that of 320 patients I have seen treated there during three months, 31 were under 15 years, 90 under 20, 80 under 25, 44 under 30, 22 under 35, 13 under 40, and above 40 were 22, of whom 13 were under 50, 5 under 60, and the remaining 4 were aged as follows—1 of 70, 1 of 73, 1 of 76, and 1 of 95 (the latter died of old age); consequently, above one third of the whole number were under 20 years, and considerably more than one half under 25 years. This circumstance of comparative youth, under all kinds of treatment, has an immense influence upon the ultimate result.

Again, the patients are admitted and discharged by the physician, without any control, so that, to say the least, it requires a man to be very conscientious to decide impartially between temporary improvement and perfect cure, especially when he recollects that the fate of his creed and his institution may depend upon the nature of his returns to government. These returns are made monthly, with a yearly *resumé*.

Some of the following cases will be found to have been discharged too early to enable us to be positive as to the ultimate result. Again, these cases, or others discharged apparently cured, may apply for readmission, and be, under some pretext or other, refused; while, to disarm suspicion, a few whose relapses seem more manageable may be readmitted. Such may not be the case in point of fact, still it is very possible. I have seen at least one patient refused admittance, and that, too, the very day after his discharge, without any good obvious reason. It was a boy, with effusion into the right pleura following scarlatina, which he had gone through at home. There was also a general anasarcous state of the body, which speedily disappeared, but the chief complaint remained obstinate, and after 33 days' treatment with bryonia, the 2d dilution, four times daily, he was dismissed but slightly improved. This boy was denied admission when he applied the following day on account of return of pain in the chest, not certainly for want of room, as his bed was empty for days afterwards. This is not the only effusion into the chest which has been dismissed unimproved during the period of my observations; yet this scarcely agrees with Dr. Fleischmann's returns, as out of 12 with exudation in the pleura, occurring during 10 years, he has, he says, cured all but 3, who died. And a physician of the General Hospital has assured me that many such cases dismissed by Dr. Fleischmann, and subsequently refused admission, have applied to him for relief, and which relief they have obtained by the use of purgatives and baths. Then again, there are, I may say, hundreds of trifling cases admitted here, which would not have been admitted into any hospital in England. Many of the patients get no medicine; a few a single dose; and even of comparatively trifling cases many remain for weeks, nay months, in the hospital; while more acute or more interesting cases are hurried out too often with the cure incomplete.

Dr. Fleischmann's usual number of drugs is not very extensive, one drug serving for a great many diseases, but chiefly because the diseases principally consist of a few standard ones constantly repeated. Gastricismus, typhus, and pneumonia are the chief; and in treating these he employs almost always the same remedies, only varying when some one unusual symptom is very predominant. This uniformity is a cause of complaint from his fellow-practitioners, who say that "by seeing his practice you merely get a glimpse of what homœopathy can do; as Dr. Fleischmann, satisfied that his returns are superior to those of any allopathic hospital, gives himself no trouble in trying to suit the remedy to the disease, but is content if occasionally the disease suits the remedy—when it produces those miraculous effects which are the boast of homœopathy."

This may be so: yet it strikes me as being something extraordinary that those cases in which such miraculous effects have been produced, have always been cases of some standing, either ill-treated or not previously treated at all, and the homœpath having come in with his dilutions at the lucky moment when Nature was going to relieve herself. This always puts me in mind of the Irish proverb, "The hour that is darkest is the hour before day." Things could not be worse; they might, and luckily do improve; and the homœopath gets the credit of it. The case is then published as one of the triumphs of homœopathy, whilst the many similar cases where even homœopathic treatment has proved unavailing, are silently passed over, or recorded as instances of the imperfection of the human intellect: the wrong remedy must have been chosen!

Dr. Fleischmann also uses cold applications to the head in delirium, sometimes in headache, and cold washing of the body in fevers; in arthritis, cloths dipped in cold water, and surrounded by oil silk, are wrapped over the affected joints, and allowed to remain so long as they are damp, and are then reapplied. He also uses for costiveness clysters of plain warm water, or mixed with a little salt. In diarrhea, rice clysters are employed. He told me that neither he nor any rational homœopath ever employed emetics or purgatives, however simple; yet I heard his assistant once order a woman to get a spoonful of oil.

The number of students allowed to attend the hospital is limited by the spiritual powers, on account of the nuns. They attend generally in the morning; but during a part of my visit several attended in the afternoon, the number in the morning being full.

The whole process of the admission and discharge of patients is mysterious. Still so much is certain, that most of those admitted have been previously visited at their own houses by the assistant. Many cases not improving, or apparently not likely to improve, are got rid of very summarily. During most of the time, I visited in the morning along with Dr. Fleischmann, and latterly, for some weeks, in the afternoon along with his assistant, it not being then permitted to visit in the morning. I was told the cause of this restriction was that the students might have an opportunity of taking a course from the assistant.

I feel convinced that the secret of Dr. Fleischmann's great seeming success lies in the fact of the admissions and dismissions being entirely uncontrolled, and there being no check on the diagnosis. Rarely other than well-marked cases have their diagnosis written on the board at their bed-head, the others being left blank, and entered in his book, of course, as he pleases.

I have visited the hospital from the 21st of March till the 5th of August daily, except for a short time at first, when I was not in very good health; but I only give you the details of the three months, May, June, and July; as at first I was not sure how I could best conform to your wishes. Three months' regular observations are better than desultory observations extending over a longer period. I have not been able to do all that might have been desired, still I think enough has been done to enable you to judge fairly of the state of things. The patients are in general so stupid that it is not easy even for a native to obtain the requisite particulars from them; of course, it is much more difficult for a foreigner. And as to previous history, they are totally unaccustomed to recollect or relate anything of the kind. If they have been previously ill, they can seldom tell you how or by whom they have been treated. The restraint belonging to the nature of the institution prevented even Dr. Fleischmann from making that examination which, in general or allopathic practice, at least, would have been thought requisite. Of course, I could do no more; I have, however, done my best to discharge my task faithfully and impartially; and in laying the following statements before you, I may say with Störk—"*Non hypotheses condo, non opiniones vendo, quod vidi scripsi.*"

TYPHUS FEVER.

During the months of May, June, and July, there were treated 32 typhus patients. Four of these still remain under treatment; and, excluding these, six died, giving a mortality of 21·4 per cent. The average age was 21·5.

Under Skoda, in the General Hospital, during the same period, there were 76 typhus patients treated, of whom 15 still remain; 19 of those died, giving a mortality of 31 per cent. The average age was 26·4. Of these patients, however, there were, as I know from actual observation, fewer trivial cases. Skoda's treatment consists in giving acids (mineral), in doses of ℈j or ℥ ss. daily; extract. graminis, or any other equally innocuous extract, by way of *placebo* for the patient; and where the diarrhea is violent, he employs tincture of opium to moderate it, generally in much the same dose as the acids. The following notes, carefully taken at the time, will give you some idea of the homœopathic cases and their treatment by Dr. Fleischmann:

Case I.—J. A., a stout young woman, admitted Tuesday, April 27th. Next day at the visit, stated that for fourteen days past she had been suffering under daily attacks of cold followed by heat; resting, however, well by the night. (*Ipecac. 3d dilution, four times daily.*) On Friday last she was seized with vomiting, which lasted some time; bowels have not been opened for the last eight days; the pulse is accelerated; the tongue coated and dry; skin hot and dry; pain on pressing the abdomen. May 1st. The tongue cracked and bleeding. May 2d. One stool; was restless and wandering during the night; sleeping quietly at the time of the visit; cheeks flushed and eyes sparkling; tongue covered with dry brown scales. 4th. The pain in abdomen is to-day more violent and aggravated by slightest pressure. 5th. Bowels opened three times; skin cooler. 6th. Bowels opened eight times; thirst great; sleeps but little; wandering a little at night; the tongue is clearing and moist; the pulse less feverish. 7th. Bowels opened three times; slept better last night; coughs a little to-day, and expectorates a quantity of dull ill-coloured mucus streaked with blood; percussion-sound is good, but sonorous râles are audible over the chest. 8th. Bowels opened six times; abdomen slightly tympanitic. 9th. Her appearance is to-day more lively and sensible; the pain in abdomen better; the diarrhea ceased; tongue moist. 12th. The tongue is again dry and covered with brown scales; bowels not opened for three days; the pulse, however, remains quiet, the skin cool; expectoration continues. 15th. The pain in the abdomen is entirely gone; bowels open to-day. 17th. On account of difficult expectoration she got senega, the second dilution, to be given alternately with the former medicine, which she got on the 28th of April, viz. ipecacuanha, the third dilution, four times daily. 19th. She is to-day bathed in perspiration; the pulse quiet; expectoration easy, and tongue again moist and clearing. 24th. Her cough is now much less troublesome. 26th. Her appetite has returned; the expectoration is much less copious, and more easily brought up. 28th. The ipecacuanha stopped, and the cough and expectoration having entirely ceased on the 18th of June, the senega was also stopped. On the 19th of June she was seized with a rheumatic attack in her ankles, which confined her to bed for a few days, after which she continued daily to improve; but was not discharged till the 26th of July.

Case II.—M. M., a delicate-looking woman, aged 35, admitted on Thursday, April 30th. Complained at visit next day of rigors followed by heat, which together last from six to ten every evening, and have occurred daily for the last three days; they are followed by wakefulness, which lasts till the morning, when she has a little broken slumber; there is constant headache and bitter taste in the mouth; tongue coated; bowels open every third day; no appetite; slight pain in abdomen on pressure. (*Bryonia, 3d dilution, four times daily.*) The rigors left her; the fever increased somewhat, the tongue drying; otherwise her state was unchanged till May 9th, when she was covered with sudamina, having perspired a good deal during the night; the tongue moist and clearing; but on the 13th it was again dry, covered with brown scales; diarrhea set in, the bowels having been twice opened, and continued more or less for three or four days. On the 20th a slight cough came on, with mucous expectoration; this was gone by the 25th; the medicine stopped on the 26th, on which day she was up, still feeling very feeble, but otherwise free from complaint. On the 2d of June she was discharged.

Case III.—T. B., a stout-looking woman, aged 26, admitted Sunday, May 3d. Next day, at visit, found her lying with flushed cheeks and wandering eyes, and a cold compress on her head; she would not speak above a whisper, saying that she was unable to do more; stated that for eight days past she had been suffering from alternate attacks of cold and heat; had no headache nor pain anywhere; was thirsty, and could not sleep; tongue coated and dry; pulse soft and accelerated. (*Belladonna, 4th dilution, three times daily.*) On the 7th she would neither speak nor take medicine, and continued lying in this stupid state till the 9th, when she seemed more sensible; her state otherwise unchanged. 12th. Has had a slight bleeding at the nose; complains of pain in the throat, but nothing can be detected on examination; she also swallows quite easily, sleeps well, speaks rationally; the pulse is quieter; the skin cool and moist; tongue clearing and moist; the feeling in the throat continued for a day or two longer, and then disappeared; she continued improving till the 31st, when she was allowed to rise, the medicine being discontinued; and on the 4th of June she was discharged, pain in the abdomen and diarrhea having been altogether absent.

Case IV.—S. T., a stout woman, aged 26, admitted Wednesday, May 6th. Stated that she had been unwell for eight days. Her tongue is dry and cracked; pulse accelerated; skin cool and moist; no pain in the abdomen. (*Bryonia, 3d dilution, four times daily.*) 9th. Covered with perspiration; tongue with dry brown scales. 15th. Slight pain in abdomen on pressure; it is also tympanitic; seemed, however, better; tongue moist; skin cool, and pulse quieter; bowels opened three times; diarrhea continued for four days. On the 21st her cheeks were flushed, pulse fuller and faster; delirium came on during the night. 22d. (*Bryonia omitted; opium, 3d dilution, three times daily.*) Tongue dry; skin hot, with an increase of the fever during the night. In this state she continued till the 27th, on which night she died; no section.

Case V.—M. E., a female, aged 31, admitted on Thursday, May 7th; next day at visit, stated that she had been ill for four days with headache and feeling of weakness; her tongue is dry and coated; pulse feverish; pain in abdomen on pressure. (*Belladonna, 3d dilution, four times daily.*) To this was added, in a few days, cough and expectoration of a dark-coloured mucus; and on May 11th, on account of difficulty of expectoration, the belladonna was alternated with senega, the 4th dilution; the percussion-sound was good; the auscultation gave mucous and sonorous râles. On the 17th of May the belladonna was omitted and arsenic given. On the 20th she expectorated a membranaceous mass about the size of a finger; the pulse is quick and weak; countenance rather sunk; face pale and lips blue; skin still hot; delirious at nights. 22d. The senega omitted, and opium, 3d dilution, four times daily, substituted. 23d. Pulse upwards of 130, feeble; thirst great; hands and arms tremble; breathing hurried. 24th. Bowels opened four times; pulse somewhat improved. 28th. Carbo substituted for arsenic: diarrhea continues; alvine discharges black; almost constantly sensible; pulse firmer; appearance somewhat improved. June 2d. The diarrhea has nearly ceased; the countenance again sunk; pulse quick and feeble; expectoration difficult; senega to be given instead of opium. 4th. Bowels still loose; looking better; slept a little. 8th. Sleeps well; cough gone; pulse quiet and stronger. 8th. Skin cool; feels altogether better. 10th. Complains of pain to-day for first time; a slough is forming over right hip, which did not, however, separate till the 20th; it was as large as a man's fist. During this time she had considerably improved, but after this she once more relapsed, gradually sinking, and died on the 28th. No section.

Case VI.—K. K., a girl, aged 18, admitted June 15th, stated that she had been ill for eight days, the illness commencing by attacks of cold and heat, accompanied by a continuous headache and pain in the abdomen; has been during this period treated homœopathically; the skin is hot and dry; the tongue dry, brown, and cracked; pulse fast, but rather feeble. (*Arsenic, 4th dilution, three times daily.*) 16th. Has been restless and feverish during night; wandering occasionally in mind; tongue and teeth covered with black sordes; her face is pale; she continued in this low delirious state, with increase of fever at night, discharging her urine and fæces involuntarily, diarrhea having set in on the 20th till the 25th, when her pulse improved, becoming quieter, and her tongue moister. On the 26th she was livelier, and answered questions more readily. 28th. She again relapsed; tongue dry; skin hot; pulse accelerated. June 1st. Pulse weak, scarcely perceptible; respiration hurried; face pale and death-like; four stools. 2d. Skin cool; respiration more regular; tongue moister; pulse quieter; her mind wanders still occasionally; the tongue now began to clear; she lost her wandering, and gradually became more lively in appearance; pulse became quiet, and gradually improved in strength—in short, from this time she steadily improved till the 26th, when she was discharged.

CASE VIII.—M. S., a female, aged 21, admitted July 20th. She was partially delirious, and unable to give any account of herself, except that she had been unwell for three weeks. The tongue is red, cracked, and bleeding; respiration difficult; pulse fast and weak; pain in abdomen on pressure; percussion-sound good; respiration vesicular, mixed with râle; sputa yellow, streaked with blood, and slightly adhering to the vessel. (*Phosphor. 3d dilution, every second hour.*) She gradually sunk, and died on the 26th. No section.

The general run of the cases here resemble the first two or three. Those like the latter seem only admitted occasionally—I suppose by way of attempting a bold stroke. Although I have at present got no more cases written out, yet I have all the severe ones noted, and the period of treatment, which I could furnish you with, if necessary. The average period of treatment was twenty-three days.

### FEBRIS GASTRICA.

There were also 23 cases of febris gastrica treated. The average period of treatment was 10·8 days; the average age 27·8; they were all trifling except the following.

CASE.—S. D., a stout boy, aged 15, has been unwell for the last five days; complains of sickness and headache, with a feeling of oppression over the stomach; the tongue is coated but moist; the pulse full, and rather fast; he was admitted on the 17th of May, and got the 3d dilution of nux (vomica?) four times daily. 19th. He has been delirious during night, and this still being the case, on the 20th he got hyoscyamus instead of nux, in the same dose and manner. May 24th. He was asleep; great meteorismus; pulse full and frequent; tongue still moist; skin hot. On the 28th of May the delirium was gone; the pulse quiet, and the skin more natural. He continued after this to improve; the medicine was not, however, stopped till the 11th of June, and he remained in hospital till the 26th of July.

### INTERMITTENT FEVER.

There were, during the three months, 41 cases of intermittent fever treated; the average age of the patients being 22·38. All but a very few received medicine at once on entering, without waiting to see whether they had a fever or not; a few had it once slightly, and it never returned. In 7 or 8 it never came on at all, and 30 had it repeatedly, the average number of attacks being 4·7. One had as many as 13; in one case the fever from a tertian became quotidian, and grew so much worse that the patient was discharged at his own request; the remedies employed were china, 2d and 3d dilutions, three or four times daily; ipecacuanha, the 1st dilution, and nux, the 3d, alternately three or four times in the course of the day; and ipecacuanha and nux alone in the 2d dilution more rarely, arsenic, the 4th, or aconite, the 3d dilution. Chinin was only twice employed each time in the second trituration.

Skoda's cases of ague at the General Hospital are treated in the following manner. Each patient, on complaining, gets some extract. centaurii or taraxaci, or some such thing, and one attack is observed—if that come about the time specified, good—the further treatment is proceeded with, if not, a second attack is waited for, so that the time about which the fever may be expected may be known; then supposing it to come at nine o'clock, the patient gets two grains of sulphate of quinine at six, at seven, and at eight—at each hour two grains. The fever, if it be a regular one, may come slightly once after this, but never oftener; so that in regular cases, three times is the oftenest, generally only twice, and in rare and irregular cases four times—never oftener; but it also very frequently remains away altogether under the use of the bitter extracts, even where it has been of six weeks' duration, as I have myself seen; the powders are continued for three or four days after the last attack, in the same manner; a recurrence of the fever during the residence in the wards has not yet been seen, though this residence is sometimes, from the nature of the hospital regulations, protracted.

To return to the Homœopathic Hospital. In one case the fever returned after three weeks' absence, during the girl's residence in the wards; after two attacks, it again left; two of the patients I have seen in the cold fit of the fever, and can testify as to its severity; and two or three I have also seen in the hot fit. A boy who had one of the severest fevers, returned after six weeks' absence, on account of bronchitis; he was looking much better, but as he spoke nothing but Bohemian, I could not interrogate him as to his fever. The following case was also put down as an intermittent:

Case.—W. M., a boy, aged 12, admitted on Sunday, June 28th. Next day, at visit, stated that he had had fever every day for the last four weeks. The attending sister stated that she had observed no cold fit the previous evening, though he had had a hot one. (*China, 2d dilution, four times daily.*) Upon a cross-examination, after Dr. Fleischmann had passed on, he said that for three weeks he had had a regular fever, since then every evening merely a hot fit. The percussion over the heart is dull from the third to between the seventh and eighth ribs. The first sound of the heart is somewhat impure; the pulse is irregular, every five or six beats, one failing, or rather in its place a faltering sort of beat is to be perceived; when lying on his back the heart's pulsations are not perceptible to the touch, though when he sits up, and leans slightly forward, they are then readily felt; this having been found and stated to Dr. Fleischmann, he declared that, on account of the absence of both pain and oppression in the breathing, he could not believe that there was any exudation present, he still believed him to have intermittent. On the 29th and 30th he was stated to have had regular fever, and said himself that he had a slight cold fit; after this he had no more. He was up on the 8th, and discharged on the 9th, the percussion-sound over the heart being unchanged, the first sound purer, and the pulse less distinctly intermittent.

### RHEUMATISM.

There have been 27 cases of rheumatism; the average age of the patients was 25. The following deserve notice:

Case I.—M. K., a young woman, aged 20, admitted on the 4th of May. The knuckles of both hands are considerably swollen and red; elbows slightly; complains of pains throughout the body and a pain in the left side; she states that she has suffered from rheumatic fever for fourteen days; there is a slight bruit with the first sound of the heart. (*Spigelia, 3d dilution, four times*

*daily.*) The arms were after this enveloped in cloths dipped in cold water and covered with oilskin; these were not changed so long as they were damp; continued complaining grievously of pain till the 9th, when a diarrhea set in, after which the swelling and redness of the joints disappeared, and the pain speedily ceased. By the 16th the slight bruit had entirely gone; the joints were quite well; the diarrhea still continuing; the spigelia was stopped and ipecacuanha given; the diarrhea did not return, and on the 23d she was discharged cured.

Case II.—J. P., aged 16, a young man, admitted Tuesday, May 12th. Said that eight days ago he had a rigor followed by a hot fit. Since then his ankles, knuckles, and wrists have been swollen, red, and painful, even on the slightest motion; he complains of pain in left chest, and has a slight cough. (*Acidum phosphoricum, 3d dilution, four times daily.*) Upon auscultation a bruit is heard with the diastole, over apex of heart. The rheumatism without further treatment was quite gone by 16th or 17th, leaving, however, a weakness in the ankles, which continued till his discharge; he is altogether a feeble-looking young man; the bruit also gradually diminished, and by the 27th was entirely gone. He was not discharged till the 10th of June.

Case III.—Another articular affection of a similar nature, occurring in a girl aged 18, and admitted on the 10th of June, of five days' standing, was treated solely by the wet compresses, and in five days she was discharged cured.

Case IV.—A. B., a delicate woman, aged 24, admitted on the 25th of June with a general rheumatic affection, of eight days' standing. She had for a year previously suffered under chronic gout and abdominal congestion, as she called it "leberkrankheit;" she continued without any medicine, and without expressing any peculiar feeling of pain till the 15th of July, when she got *Rhododendron, 3d dilution, three times daily.* The pain has settled in the left knee, and almost entirely left the rest of the body; she complained of the most excruciating agony there, and continued to do so till the commencement of August, when I left. The last two or three nights she had not closed an eye, she was rapidly emaciating, and the attending sister said that her knee was much swollen, yet no examination was made.

Case V.—K. B., a healthy-looking young woman, aged 24, admitted June 28th. Stated that a year ago she had had an attack of rheumatic fever, and since then had never been entirely free from occasional twinges; that eight days ago, on exposure to cold, the pains in her knuckles, wrists, and ankles had returned, accompanied by oppression in the chest, and occasional fits of difficult respiration; the affected parts are not the least swollen, indeed she can move them pretty freely; over the heart a bruit is heard at the apex along with the first sound; second sound is natural, but above it in the aorta is heard synchronous with the systole a loud rasping noise; her menstruation is quite correct; she has had a blister on the chest. (*Spigelia, 3d dilution, four times daily.*) The fits of dyspnea recurred for four or five days in the evening, and then left; the first sound of the heart became purer, the rasping sound in the aorta still continued; the pulse was generally quiet, and of tolerable strength, but she was much troubled with palpitation, generally recurring about noon. On the 9th of July her hands and ankles were swollen and painful, and continued so till the 13th, when the swelling again vanished; neither the appearance nor disappearance of this had any effect on the general symptoms. About the 29th of July rheumatic headaches made their appearance, and gradually increased in severity till she was discharged on the 3d of August. The sound in the aorta the same as before. The first sound still impure, the ankles weak and painful, the palpitations recurring daily.

The following case had also "Rheumatism" written as its diagnosis:

Case VI.—J. P., a young man, aged 19, complains of pain along the region of the spine; the inferior extremities are nearly completely paralysed; whenever he attempts to move them he only produces slight convulsive twitches; an enlarged gland is situated in the right submaxillary region. (*Dulcamara, 3d dilution, twice daily.*) The urine flows off spontaneously; the bladder on percussion is found to be quite distended, and in this state it remained during his residence. On the 28th of June the dulcamara was omitted, and *nux, 1st dilution, four times daily* given. He had two or three attacks of intermittent, and ipecacuanha was added on the 31st; this did not recur. On the 1st of July, having had no motion for eight days, and the abdomen being quite tympanitic, a clyster was given. After this nothing occurred; he gradually sunk and died on the 28th of July, sores having formed on his œdematous feet.

The other cases of rheumatism were pleurodynia, or slight muscular pains. The two following cases remained in the hospital when I left:

Case VII.—A girl, aged 16, admitted on the 31st of July. She had been ill with rheumatic fever for three weeks. The right elbow and shoulder and left ankle were swollen, red, and painful; there was no heart affection. She got aconite, 2d dilution, every second hour.

Case VIII.—A girl, A. F., aged 23, and admitted on the 27th of July, had been ill five days with a rheumatic attack in the muscles of the neck, and subject to daily exacerbation in the afternoon. However, on the afternoon of her admission this had not taken place, consequently could not be attributed to medicine, which she had not got. She improved steadily, and though well was not discharged when I left. She had aconite, 3d dilution, four times daily.

### DIARRHEA.

There were eleven cases of simple diarrhea treated: average age 26·3 The medicines employed were acidum phosphoricum, 2d and 3d dilutions; ipecacuanha, 3d dilution, four times daily. In no case was the diarrhea checked or even moderated on giving the medicine, but in the course of a few days it gradually ceased, though frequently with previous exacerbations.

### DYSENTERY.

Three cases of dysentery occurred.

Case I.—A female, aged 31, admitted on Friday, May 8th. Has been unwell for eight days; yesterday the stools were, for the first time, accompanied by blood and straining; no thirst nor pain in abdomen; tongue coated but moist; pulse very little accelerated. (*Sublimat., 3d dilution, four times daily.*) The stools continued five or six times daily, mixed with blood and mucus till the 12th of May, when they became more natural; ceased altogether on the 13th. The medicine was stopped on the 15th, and she was discharged on the 18th.

Case II.—A man, aged 28, who had been unwell for three or four days; his stools on admission, the 29th of April, were chiefly blood and mucus, accompanied by straining and pain in abdomen on pressure; they amounted to 40 in the course of the day. (*Sublimat., the 3d dilution, four times daily.*) His pulse is slightly accelerated; skin natural; tongue moist; the fever increased slightly for a day or two, the skin becoming hot, and the tongue dry, and again relaxed, both returning to their natural condition. On the 7th of May the stools had diminished to eighteen. *Sublimat.* removed, and *rhus, 3d dilution, three times daily,* substituted; to which *ipecacuanha, the 2d dilution, four times daily,* was added on the 9th. By the 12th the stools were more natural, and by the 14th had entirely ceased. He remained in hospital, having been much reduced till the 4th of June, when he was discharged.

Case. III.—A. S., a woman, aged 45, admitted July 14th. Stated that for eight days she had been afflicted with bowel complaint, having had thirty stools in the course of each day. Each stool consisted of watery mucus, mixed with blood, and was accompanied by straining and griping. She has headache, feverishness, and sweats much, sleeping little; the pulse is full and accelerated; the tongue coated and moist. (*Sublimat., 3d dilution, every third hour.*) 15th. Has had only one stool since admission, and that one without blood, free from pain, and more natural; slight pain in abdomen on pressure. Her bowels were not again opened till the day before dismissal, when she had a natural stool; the pain in abdomen had entirely disappeared. She was dismissed on the 20th of July.

### CHOLERA.

Two cases resembling cholera sporadica were treated.

Case I.—F. F., a man, aged 26, admitted Sunday, July 12th. Has always previously been healthy till Tuesday last, after eating a "saures rindfleisch," which is meat two or three days after its first cooking, rewarmed with a sour sauce, and which, I have been assured, very frequently produces the same symptoms, being a slight poison. Since then he has been almost constantly purging and vomiting; has vomited twice to-day greenish matter; stools to-day have been seven, watery, and forcibly expelled; he has headache, pain in the abdomen, cramp in the hands and feet; frequent eructations and great thirst; the tongue is red and dry; pulse fast and feeble; voice low and faint. (*Veratrum, 2d dilution, every second hour.*) 13th. Feels better; has not vomited; only twice had stools since yesterday; two attacks of cramp in the hands during night; his pulse is a little fuller, and the voice improved. 14th. Bowels again twice opened; no cramps; tongue moist, clean; pulse full and quiet; appetite returned. 15th. No pain anywhere, nor cramps; bowels opened twice. 17th. Medicine omitted, and on the 20th he was discharged.

The second case, with somewhat similar symptoms, was admitted on the 31st of July, and was left in treatment. The patient (a woman) was not, however, improving so rapidly.

### COLICA.

There were three cases of colic.

Case I.—This was a simple colic; ceased in a few days under the use of *opium, the 6th dilution, three times daily.*

Case II.—This a colica menstrualis. The patient got *cocculus, 6th dilution, twice daily;* the third day became furiously delirious, for which she got *stramonium, 3d dilution, four times daily,* and was then removed into a private room, after which I never saw her.

Case III.—H. B., a stout man, of 42, admitted Thursday, July 16th. He stated that he had been for years a painter; had previously had colic four or five times, for which he had been treated in the General Hospital here, and this was his first trial of homœopathy. He had intermitting pain in the abdomen, twisting about the umbilicus, and relieved by pressure; obstinate constipation; cramps in the calves of the legs and arms; loss of power in the hands; the edge of the gums where they join the teeth is of a blue colour; pulse quiet. (*Opium, 1st dilution, foar times daily.*) 17th. During the night he got a clyster, which brought away some fæcal matter, and he is now easier. 18th. The pain has returned somewhat; he again got a clyster, which again brought away some fæcal matter, and afforded some relief. On the 20th the attack was again worse, but it had got better spontaneously by the 21st. He says himself that it is his worst attack, and that he has been more speedily relieved than at any former period. After this the pain and cramps remained entirely away, his hands were restored to their wonted condition, but his bowels were not opened since the 18th till the 27th, when they spontaneously and copiously relieved themselves. He was discharged on the 28th.

### SCARLATINA.

Two cases of scarlatina were treated.

Case I.—J. R., aged 12, admitted July 9th. The next day, at visit, he was lying with his eyes shut, in a half comatose state; was stated to have been very restless during the night, and was indeed bound to the bed. On being aroused, which he readily was, though he speedily relapsed, he stated that since Sunday he had been unwell with pain in the abdomen and throat. His whole skin is of a bright scarlet colour, which disappears on pressure, returning from the circumference; his tongue red and dry; throat is scarcely to be seen, on account of his unwillingness to open his mouth, and when it is forced open he complains of pain most grievously; it is internally much swollen and red; no swelling externally; he has had a few leeches on his throat; pulse very feverish; skin hot. (*Belladonna, 1st dilution, every second hour.*) 11th. An eruption like sudamina is scattered over the chest; he has been more restless than ever during the night; the comatose state still continued. The fever increasing, he died on the evening of the 12th. No section.

Case II.—J. K., a boy, aged 7, admitted Sunday, July 26th. Next day, at visit, he complained of sore throat, but was unable to state how long he had been ill. Skin covered with a red rash, which disappears on pressure, returning from the circumference, and in points; he has on a thick cotton night-shirt, beneath that several cloths; his hands and feet enveloped in stockings, so that nothing but his face is left uncovered; he will not put out his tongue; his pulse is very slightly accelerated, for a boy of his age perhaps not at all. (*Belladonna, 3d dilution, every third hour.*) 29th. Tongue red and dryish; eruption beginning to fade. 30th. The cuticle beginning to scale off; his pulse is full and slow. 31st. Sitting up; a slight ulcer has formed on the tip of the tongue; medicine discontinued; his stockings, &c. are discarded; I left him running about the ward quite well.

### PNEUMONIA.

Case I.—F. H., a stout-looking young man, aged 22, admitted on the 5th of May. Next day, at visit, stated that five days previously, after a sudden cooling, he had been attacked by alternating fits of cold and heat, followed by pain in the chest and cough; the two latter symptoms have since been constant, though the cough has not been very troublesome; he has, however, during the night expectorated a considerable quantity of a dark bloody-looking fluid, which still continues to be brought up in less quantity, mixed with mucus and saliva; his countenance is extremely anxious; his respiration hurried and imperfectly performed; his pulse full, but extremely rapid; on percussion the sound on the right side anteriorly is good as low down as the centre of the mammary space; on the left the same, with the exception of a space of two and a half inches from the left edge of the sternum and from the fourth rib; this space, and all beneath on both sides, are dull; this dulness posteriorly extends as high as the centre of the scapula and intrascapular spaces on both sides; the respiration on both sides, over the dull portions, is pure bronchial; over the superior portions of both lungs vesicular; though, from the above-mentioned state of the respiration, not always very distinct. (*The 3d dilution of phosphorus was ordered to be given every second hour.*) The patient died on the afternoon of the 6th.

Section, Friday, 8th, 10 a.m.—On opening the thorax both lungs were found firmly connected by cellular adhesions to the parietal pleura, particularly their inferior lobes; over the superior and external portion of the right lung was a small, soft, albuminous-looking exudation, of about half an inch thick; the inferior lobe of the left lung and the inferior and middle lobes of the right one were in a state of red hepatization, soft, and easily broken down; a considerable quantity of reddish serosity gushed from their cut surfaces; the superior lobes of both lungs contained a quantity of frothy mucus; the cavities of the heart and large vessels contained a small soft clot; the intestines were gorged with blood, and the glands of the ileum slightly swollen. The other organs were not examined, except the spleen, which was soft, but scarcely enlarged.

Case II.—F. H., a healthy-looking boy, of 16 years, admitted on the morning of Saturday, May 9th. At visit, same day, stated that two days ago he had a rigor followed by a hot fit, accompanied by perspiration, and followed by pain in the left breast and cough, which were, however, so slight that he came in only on account of the feverishness which still continued. He has always previously been healthy, and never subject to cough; his cheeks are flushed; tongue somewhat coated, but moist; pulse full and accelerated; sputa frothy mucus streaked with blood; percussion-sound normal; respiration vesicular, marked, however, on the posterior portion of the left side, from the lower third of the scapular space downwards by a fine crepitating râle. (*Aconite, the 4th dilution, every third hour.*) 10th. Where crepitation was yesterday audible, bronchial respiration is to-day to be heard; the percussion-sound is also over the same space dull, anteriorly; from the lower portion of the mammary space downwards on the left side slightly tympanitic, otherwise normal; the sputa are now rusty, and slightly adhesive. 11th. The pulse is to-day much quieter; the physical signs remain the same. This state continued till the 13th, when over the superior portion of the dorsal space a crepitating râle was audible, accompanied by bronchial expiration; in the lower portion of the scapular space the respiration is still pure bronchial; dullness on percussion the same; right side normal in every respect. 14th. The dullness or percussion is to-day but slightly perceptible; the respiration is everywhere vesicular; over that portion of the left lung where bronchial respiration was formerly heard it is a little rougher than elsewhere, and accompanied by an occasional mucous râle. He has been a little restless and wandering in mind a little during the night; quite calm and collected now; has also had a slight bleeding from the nose; expectoration mucous; skin cool; pulse quiet and regular; tongue moist and clean. May 15th. Has again had a slight bleeding from the nose; percussion-sound everywhere normal; auscultation gave vesicular respiration, with a few mucous râles; the cough pains him slightly, and he has a general feeling of soreness over the chest; appetite has returned; his expectoration is mucous, and but slight; he continued improving, the râles not having been again heard; and on the 18th was allowed to get up, and discharged on the 21st.

Case III.—F. D., a slightly-made boy of 16, with a clear skin and delicate complexion, admitted on Saturday, May 16th. Stated that he had been unwell and feverish since the Thursday previous, with slight cough and pain in the chest; he had formerly been subject to cough; percussion-sound over the right lung posteriorly was dull as far down as the centre of scapular space; anteriorly over the same lung tympanitic, as low as the commencement of the mammary region; over the rest of the chest normal; the respiration was found on auscultation everywhere vesicular, with the exception of the above-mentioned dull space, where it was bronchial; tongue moist and coated; pulse full and accelerated, (*The 4th dilution of phosphorus to be given every third hour.*) In this state he continued, the pulse remaining firm and full; the skin hot and dry, and the inflammation progressing daily till the 20th; when the whole of the right side posteriorly was found dull on percussion, and the upper portion of the left, as low down as the middle of the scapular space; anteriorly, over the entire right side, the sound is tympanitic; over the left side good, with the exception of the subclavicular space, where it was somewhat duller. On auscultation, pure bronchial respiration was heard over the entire right side posteriorly, and the superior portion of the left side posteriorly; elsewhere the respiration was vesicular, and, though somewhat quickened, not laboured; the sputa were rusty and adhesive; the skin hot and dry; the tongue coated and dry; pulse full and bounding. 21st. On the left side, posteriorly, the dulness and bronchial respiration to-day extends as far down as the lower third of the scapular space; the other physical signs remain unchanged; the skin is cool; pulse quiet, and the tongue moist. 22d. The percussion remaining the same; the sounds, on auscultation, are so far altered, that over the acromial and upper third of the scapular space, on the right side, instead of pure bronchial, an undecided respiration is heard. May 23d. The physical signs the same as yesterday, with the addition of a slight crepitating râle occasionally audible over the right acromial and upper portion of the scapular region; the patient is looking livelier; his skin cool; pulse quiet; expectoration looser, and more catarrhal. 24th. Percussion as formerly; the respiration over the entire right side, posteriorly, was sharp, vesicular, puerile, mingled with occasional fine crepitating râles; anteriorly, on both sides, vesicular; posteriorly, on the left, undecided; marked bronchial as low as the top of the middle third of scapular space; the middle third bronchial; beneath vesicular; expectoration colourless mucus. 25th. Vesicular respiration everywhere to be heard, a little rougher in character over the middle third of scapular space of the left side; where the bronchial respiration was yesterday audible, no râle is to-day audible. 26th. Physical signs as yesterday; the dulness on the left side perhaps a little less distinct, and a few subcrepitant râles are heard over the right posterior dorsal space; cough is not so troublesome; expectoration easy, mucous. 27th. Left acromial and scapular regions decidedly clearer; no râle audible; respiration everywhere vesicular. 28th. Entire left side clear on percussion; otherwise no change. 29th. No change. 30th. Dulness over right side; posteriorly, less in intensity; anteriorly, normal; left side normal; numerous mucous râles are to-day mixed with the vesicular respiration; the expectoration and cough are somewhat increased. (*Medicine to be given only thrice a day.*) The patient continued daily to improve, and on the 4th of June there was only an occasional mucous râle audible over the right dorsal region; and on the 6th full, clear, vesicular respiration everywhere audible; the chest expanding well, and the cough gone; but still the percussion slightly dull, though improved, on the right side posteriorly; after this, the patient was always up and dressed during visit, and was discharged on the 11th of June.

Case IV.—L. U., a stout healthy-looking young man, aged 18, admitted on Saturday, the 16th of May. Stated at visit, next day, that he had been on the previous Wednesday seized with pain in the chest and cough, which continue. Percussion-sound anteriorly over left side normal; over right side tympanitic, as low as commencement of mammary space; posteriorly, left side, normal; right side dull down to centre of scapular space; beneath normal; respiration everywhere vesicular, except over dull space on the right side posteriorly, where it was bronchial; sputa rusty, adhesive; pulse quick and full. (*The 3d dilution of phosphorus to be given four times daily.*) May 18th. Physical signs the same; skin covered with profuse perspiration; bowels opened six times. 19th. Bowels to-day not opened; on auscultation, where the bronchial respiration was audible, it is now indistinct and mixed with a fine crepitating râle; pulse quiet and regular; expectoration mucous. 20th. Respiration to-day everywhere vesicular; the crepitant râle still audible in the same situation as yesterday. 21st. The percussion-sound over affected part is to-day somewhat clearer; condition otherwise the same. 22d. Percussion-sound still improving, and on the 23d everywhere good; the vesicular respiration is everywhere audible, and free from admixture of râle; cough gone; after this the patient remained in hospital free from all complaint till the 28th, when he was discharged.

Case V.—E. B., a stout-looking woman, aged 48, admitted Saturday, May 16th, afflicted with pain in chest and difficulty of breathing. She got *the 3d dilution of aconite, four times daily;* exchanged on the 20th for *the 3d dilution of phosphor., four times daily,* and being on the 21st evidently much worse, mind excited and wandering; expectoration retained. *Tartarus emeticus* was given in the 2d dilution, three times daily. The patient died during the day.

Section, Saturday, May 23d, 10 a.m.—Extensive plastic exudation was found between the visceral and parietal portions of left pleura, forming at the lower portion a sac containing fluid; the entire inferior lobe of left lung in a state of gray hepatization; right lung and upper lobe of left, though not to the same extent, gorged with blood and serum; bronchi filled with frothy mucus; right lung connected to costal pleura by old cellular adhesions; a small amount of plastic exudation in the pericardium; the right heart contained a fibrous clot, the left one was empty; other viscera, so far as examined, normal.

Case VI.—A. T., a stout-looking man, aged 46, admitted Thursday, May 21st. Same day, at visit, stated that he had caught cold, and had now great pain in breathing, accompanied by a slight cough; he has not expectorated since admission; percussion everywhere normal; respiration vesicular; pulse full, strong, and accelerated. (*Aconite, the 3d dilution, four times daily.*) May 22d. Expectoration rusty, blood-stained; percussion anteriorly on left side normal; on right side dull as high as centre of mammary space; posteriorly, on left side, normal; on right side dull as high as centre of scapular space; dulness likewise extends round over the lateral and infralateral spaces; elsewhere vesicular; pulse still full and bounding; *aconite* stopped. and *phosphor.* given, the 3d dilution, four times daily. 23d. Not examined, on account of his evidently dying condition; the breathing hurriedly and imperfectly performed; the countenance extremely anxious; pulse still more accelerated, but failing in firmness and strength. He died in the course of the day. No section was made.

Case VII.—F. B., a young woman, aged 24, admitted Sunday, May 24th. Next day, at visit, she stated that, having been always previously healthy, she had five days ago caught cold, and since then been suffering from pains in the chest, accompanied by cough, most troublesome at night. Expectoration slight, rusty, and adherent; has no headache, and no appetite, but great thirst; percussion-sound good, except posteriorly over right lung, as far down as centre of scapular space; beneath this again good; on auscultation, mucous râles are heard pretty generally distributed over both lungs; over the dull space bronchial respiration is audible; pulse accelerated; tongue coated, inclined to be dry. (*Phosphor. 2d dilution, three times daily.*) 26th. Physical signs the same; the râles, however, are not audible to-day; the tongue is moister, pulse quieter. 27th. The expectoration more mucous, less adhesive, still blood-stained; respiration undecided over dull portion. 28th. Respiration everywhere vesicular; over the dull portion rougher; more puerile; expectoration mucous; the dulness is also a shade better. After this she continued improving; by the 31st her cough was entirely gone; the medicine was, however, not discontinued till the 2d of June, and she was discharged on the 4th with still a shade of dulness over the right side, posteriorly extending, however, no farther than the top of the scapular space.

Case VIII.—A. S., a young man, aged 17, admitted Wednesday, May 27th. Next day, at visit, stated that the previous Sunday he had fallen suddenly to the ground without known cause; had always previously been healthy. Has since then been troubled with headache, sleeplessness, slight cough, and pain in the chest; he feels weak, and is tormented by thirst; his tongue is red, but moist; skin natural, and pulse but slightly accelerated; the percussion-sound is anteriorly normal, with the exception of over the right clavicle, where it is slightly dull, and where a fine mucous râle is to be heard; posteriorly, on the left side, normal; on the right one also good, down to nearly the inferior border of the scapular space, beneath which it is dull; the respiration is vesicular, obscured posteriorly on the right side by loud, sonorous, and sibilant râles; expectoration, a jelly-like mucus. (*Phosphor. 4th dilution, every third hour.*) 29th. Face to-day has a dingy appearance, with a dusky red spot on each cheek; pulse more accelerated; sputa adherent, of a deep orange colour; percussion as yesterday; pure bronchial respiration over the posterior inferior portion of the right chest, mentioned yesterday as dull; superiorly, still an occasional sonorous râle to be heard. 30th. The dulness on percussion extends now half across the inferior portion of the lateral and infralateral spaces; anteriorly, the right infra-mammary and inferior portion of the mammary are tympanitic, the rest normal; bronchial respiration is audible over the dull portions; sonorous râles are now more audible over the right anterior portion of the chest. 31st. Posteriorly, the percussion-sound remains the same; the portion anteriorly, which gave yesterday a tympanitic sound, gives to-day a slightly dull sound; where bronchial respiration was yesterday audible, the respiration is to-day indistinct, and accompanied by consonating râle. June 1st. The percussion remaining the same, a fine, loud, consonating râle is heard over the dull spaces, both anteriorly and posteriorly on the right side; the left side still gives only vesicular respiration; the skin is still dingy; the sputa less yellow and more copious; the pulse quieter. 2d. The percussion unaltered; the râles less frequent; the expectoration more copious, and now of gray mucus; the patient sleeps better, and the colour of his skin has decidedly improved. 3d. Percussion, posteriorly and laterally, as formerly; anteriorly normal, except over right clavicle, where dulness still continues; auscultation as formerly; expectoration grayish mucus; tongue moist and natural; skin cool, pulse quiet. 4th. A few consonating râles still to be heard, mixed with an undecided respiration. 6th. Percussion good, excepting, as before, over the right clavicle, and also the right infralateral and inferior portion of lateral region, where it is still a little dull; the respiration is also there undecided, elsewhere vesicular; somewhat puerile over the posterior inferior portion of right chest. On the 8th, the percussion and respiration being everywhere normal, except over right clavicle where slight dulness still continues; the medicine was discontinued; the expectoration slight; cough almost gone. On the 10th he was up, and on the 15th discharged, both having entirely ceased.

CASE IX.—M. U., a young woman, aged 19, admitted on Thursday, May 28th. Stated next day, at visit, that on the Sunday previous she had been seized with shivering, followed by heat, pain in the chest, and cough. Has been previously subject to cough; expectoration tolerably copious, adherent, and blood-stained; percussion normal, except posteriorly, on the right side, where it is dull as low down as the centre of the scapular space; beneath that normal, over the dull portion bronchial respiration is heard; over the rest of the chest sonorous and sibilant râles. (*Phosphor. the 2d dilution, to be given every second hour.*) 30th. Expectoration more copious; cough more troublesome; physical signs unchanged. 31st. Breathing laboured and anxious; entire right side, posteriorly, dull; anteriorly also; percussion-sound less clear than on the left; posteriorly, over the right chest, bronchial respiration was heard, mixed with consonating râle; over the left side, sonorous and sibilant râles; anteriorly, over both sides, loud, sonorous, and sibilant râles to be heard; expectoration still copious; pulse full and accelerated. June 1st. Physical signs unchanged; breathing less oppressed, and her look less anxious. 2d. The râles are to-day not so frequent. 3d. They are to-day much louder, especially anteriorly, and quite perceptible to the touch, i. e the thrill caused by them is so; and the respiration is again more laboured; the pulse is, however, not so feverish. 4th and 5th. Her condition continued to improve during these two days; the expectoration becoming more copious, and more free from blood-stain; and on the 6th, there were anteriorly no more râles to be heard; the percussion, anteriorly, is normal; posteriorly, the right side is still dull; the respiration there is, superiorly, undecided; inferiorly, disguised by sonorous râles; a few of these are also to be heard over the left chest posteriorly; the expectoration is copious, gray frothy mucus; the cough is troublesome. (*Medicine to be given only twice daily.*) 7th. Except an improvement in the percussion-sound of right chest, her state is unaltered. 8th. Percussion everywhere good; respiration vesicular; disguised, posteriorly, on the right side, by sonorous râles. 10th. Vesicular murmur to-day everywhere audible; cough greatly better; expectoration more easily brought up, less copious. On the 11th she was allowed to rise; and was discharged on the 13th, her cough having entirely ceased.

CASE X.—M. B., a stout healthy-looking man, aged 58, admitted Tuesday, June 9th. On the morning of the 10th he was going about the ward, and was not noticed till the end of the visit, when there was no time to examine him; no medicine was ordered. On the 11th, he stated that he had come in the instant he felt himself unwell; he complained of pain in the chest and cough; the expectoration copious, adherent, of an orange tint; pulse full, but slightly accelerated; percussion, anteriorly, over the left side, tympanitic; over the right normal; posteriorly, the entire left side dull; the right one normal; respiration vesicular, except posteriorly, over the left side, where nothing is audible but a loud consonating râle. (*Phosphor. 2d dilution, every third hour.*) This state remained unchanged till the morning of the 15th, when the percussion-sound remaining the same; instead of the consonating râle formerly heard, a fine crepitating one is to-day audible over entire posterior surface of left chest; the expectoration is copious, mucous; the pain very much better. 16th. The respiration is to-day vesicular, a few crepitating râles being still audible over their yesterday's seat; the percussion-sound is also less manifestly dull; the expectoration less copious, more easily brought up, and cough less troublesome. He gradually improved till the 21st, when the percussion-sound was normal, and the respiration vesicular over the entire chest; a full inspiration still, however, produces a subcrepitating râle over the former seat of disease; and this continued to be the case till the 2d of July, the patient having been up and going about since the 22d of June: on the 5th of July he was discharged.

CASE XI.—W. B., a boy of 15 years of age, admitted Friday, June 12th. On the 13th, at visit, stated that for four days he had been troubled with a cough and severe pain in the left side. Expectoration rusty and adherent; pulse but little accelerated; the percussion-sound normal, except at the under portion of left chest, where it is dull anteriorly as high as the centre of the mammary region, and posteriorly as high as the centre of scapular region; the dulness extends entirely round, through the lateral and infralateral regions; the respiration is over the dull portion bronchial, elsewhere vesicular; the heart's tones are clear and unaltered. (*Phosphor. 3d dilution, every third hour.*) His condition remained unchanged, his nose having bled slightly on the 16th till the 17th, when, the percussion being the same, the respiration is over the dull portion undecided, mixed with mucous râle; bronchophony still continues; expectoration mucous. 18th. Again a bleeding from the nose during night. 19th. The percussion-sound is to-day somewhat clearer; mucous râle still audible, and, accompanying the respiratory movements, a fine rasping sound is to-day for the first time audible, about the lower edge of the scapular space. 20th. The rasping sound is to-day almost inaudible; the respiration vesicular, with a few mucous râles; the percussion is everywhere normal; the expectoration trifling, and the cough almost gone; he is anxious to get up. 21st. Up and dressed; chest expanding fully, and without pain. On the 25th the cough having entirely disappeared, he was discharged.

Case XII.—A. S., a delicate-looking young man, aged 25 years, admitted Sunday, June 12th. Next day, at visit, stated that for the last day or two he had lost his appetite; had pain in the left side; feverish pulse; no cough; tongue coated, and a slight griping diarrhea, bowels having been opened three or four times daily; he had been treated in this hospital December previous for pneumonia; the percussion was normal, as also the respiration. (*Ipecacuanha, 3d dilution, four times daily.*) The same afternoon, pneumonia having manifested itself, he got from the assistant, *Phospor. 3d dilution, every third hour,* the preceding medicine being discontinued. 14th and 15th. No visit, in consequence of religious feasts. 16th. Expectoration copious, partly rusty, partly of an orange tint; percussion everywhere normal, except posteriorly over the inferior portion of the left chest, where it is dull as high as the inferior border of the scapular region; over this dull portion the respiration bronchial, elsewhere vesicular; pulse feverish; skin hot and dry; respiration somewhat laboured. 17th. The percussion remaining the same; the respiration is to-day undecided; the pulse quieter; the expectoration more mucous, blood-streaked; the breathing more easily performed. 18th. The dulness to-day extends as high as the centre of the scapular region on the left posterior surface of the chest; elsewhere it is normal; over the superior portion of this dull space the respiration is undecided for a short distance down, perhaps the distance of two ribs with the intervening space; next to this comes a layer of pure bronchial respiration of equal extent, and beneath, the respiration becomes again undecided; over the other parts of the chest the respiration is vesicular; the sputa are mucous, and more easily brought up; cough less troublesome, and unattended with pain. 19th. The percussion the same; over the superior portion of left dorsal region a fine crepitating râle is audible; immediately above this a layer of undecided respiration, and above this, up to the centre of left scapular region, the respiration is bronchial; elsewhere vesicular. The crepitation gradually progressed over the diseased portion, till by the 23d it occupied it entirely; the sound, on percussion, had also gradually improved, and was now almost normal. On the 24th, he was up and dressed; cough infrequent; still feels a slight uneasiness in left chest, but is able to inspire fully without pain. On the 27th he was discharged.

Case XIII.—A. L., a stout boy of 14 years, admitted on Sunday, June 28th. Next day, at visit, stated that he had caught cold on the previous Friday, after bathing, and on the Saturday felt himself unwell, with pain in the chest and cough, to which he had never been subject; the expectoration is rusty and adherent; the percussion, posteriorly, on the right side, is dull as high as the top of the lower third of the scapular region; anteriorly, the sound is on the same side tympanitic, up to about the centre of the mammary region, elsewhere normal; the respiration is vesicular, except over the dull portion, where a fine crepitating râle is heard, followed by bronchial expiration; the pulse is full and accelerated; skin hot and dry; tongue moist. (*Phosphor. the 3d dilution, four times daily.*) 30th. The dulness on percussion extends posteriorly as high as the centre of the scapular region; laterally the infralateral and lower half of the lateral region are dull; anteriorly, the dulness extends nearly as high as the centre of the mammary space above; for the distance of rather more than one intercostal space the sound is tympanitic, gradually fading into the normal; elsewhere it is normal; the respiration over the dull portion posteriorly is bronchial; anteriorly and elsewhere vesicular; in the lateral region the one passes into the other by means of an intervening undecided respiration. July 1st. Posteriorly, from the centre of the scapular region to the top of it, the sound is to-day tympanitic, otherwise as yesterday; the bronchial respiration extends now as high as the top of the scapular region; anteriorly and inferiorly over the dull portion there is no respiration audible; the expectoration is more copious, looking like a dark brown jelly; tongue moist; pulse quiet, and skin more cool. 2d. Physical signs as before, with the addition of a friction-sound at the lower border of the right scapular region; laterally it is inaudible, but anteriorly it is again met with about the centre of the mammary space, where the respiration on auscultating from above begins to vanish; the expectoration is still rusty and blood-streaked. 3d. Expectoration copious, more catarrhal, being simple mucous, with a few blood-streaks; posteriorly a mucous râle, by which the friction-sound is marked, anteriorly it is still audible; otherwise condition the same. 4th. Percussion-sound is posteriorly a little clearer; on auscultation there, a cooing sonorous râle is first heard, followed by a fine crepitating râle; anteriorly the friction-sound has disappeared; expectoration still copious, mucous. 5th. Percussion-sound posteriorly good, anteriorly beginning to clear; posteriorly the respiration is vesicular, with a few crepitating râles. He continued to improve till the 9th, when the vesicular respiration anteriorly began to be heard; the percussion-sound being much improved, but not entirely normal; a little crepitation is still to be heard posteriorly; cough and expectoration slight. 10th. The vesicular respiration is everywhere restored. 11th. The percussion is to-day everywhere normal; the sole remnant of disease being a few crepitating râles still audible on the right, posteriorly and inferiorly. On the 12th he was discharged.

Case XIV.—E. B., a delicate-looking woman, aged 20, of a clear complexion and nervous temperament, admitted on Sunday, June 21st. On the Monday following, at visit, she stated that she had been unwell for about four days, with feverishness and pain in the chest. She had for some weeks latterly been troubled with a cough, which had within a short time almost entirely ceased, but when it did come, pained her more than ever. The expectoration is rusty and scanty; skin natural, and pulse but slightly accelerated; percussion, anteriorly, dull over right subclavian space, where even the slightest pressure pains her, elsewhere normal; posteriorly, on the right side superiorly dull as far down as the centre of the scapular space, elsewhere normal; respiration is anteriorly vesicular; posteriorly, over the dull space bronchial; beneath it on the right side are numerous mucous râles, on the left vesicular. (*Phosphor.* 3*d dilution, four times daily*.) 23d. Percussion as yesterday; to-day, over the dull superior portion of the right scapular region a fine crepitation is heard on inspiration, followed by bronchial expiration; the mucous râle is to-day audible anteriorly as well as posteriorly; the expectoration is still rusty, adherent, and scanty. 24th. State the same. 25th. Pain much relieved; percussion unchanged; posteriorly, over dull portion crepitant râle followed by bronchial expiration; anteriorly, superiorly over dull portion consonating mucous râle; inferiorly anteriorly, and posteriorly on the right, mucous râle; expectoration more mucous, still scanty. 26th. Percussion anteriorly as before; posteriorly the dulness extends over rather more than two thirds of the right scapular and interscapular spaces, including also the acromial; on auscultation a consonating mucous râle is heard posteriorly over dull space; inferiorly on the right mucous râle, which is also heard anteriorly, and occasionally over the left side; expectoration more copious, frothy, mucous, mixed with rusty and yellow tougher pieces; tongue red, but moist; pulse more accelerated, rather feeble; skin hot and dry. 27th. The dulness and consonant râle extend to-day over entire right scapular and interscapular spaces; anteriorly the percussion over the superior portion of mammary space is slightly tympanitic, otherwise as before; the auscultation on the right as yesterday; on the left side the quantity of mucous râle is greatly increased, and the thrill caused by it quite perceptible to the touch; tongue posteriorly coated with a red point, inclined to be dry; pulse small and accelerated; expectoration as yesterday, but not so copious; troubled

LIBRARY OF THE
Orleans Parish Medical Society with disagreeable dreams and startings from sleep by night. 28th. Physical signs much the same; expectoration more copious, thin, frothy, mucous; pulse not so fast, and scarcely so thready in character; the skin is cooler and moist; the tongue moist. 29th. Percussion as before; over the lower half of the right scapular and interscapular region a crepitating râle is audible; superiorly a consonating, and inferiorly a non-consonating mucous râle; a quantity of mucous râle audible anteriorly and over the entire left side; the expectoration copious and mucous. 30th. Still improving; skin cool. July 1st. The dullness is now clearing off; posteriorly, on the right side mucous râle without consonance; anteriorly and over the entire left side nothing audible but vesicular respiration; expectoration still copious. 2d. As yesterday, with exception of a fine friction-sound to be heard about the centre of the right scapular space. She continued improving in every respect till the 5th, when the dullness anteriorly was quite gone, posteriorly but slightly perceptible; a fine crepitating râle is heard over the top of the right scapular space; the friction-sound, is gone, having only been audible for two days; the respiration is everywhere vesicular. 7th. The chest sounds everywhere normally on percussion; the respiration is everywhere vesicular, accompanied over the superior portion of the right dorsal region by a few mucous râles, which, by the 9th, had, along with the cough and expectoration, entirely disappeared. The medicine was then stopped. She was allowed to sit up for half an hour daily, and continued gaining rapidly in strength till the 12th, when she was discharged.

Case XV.—A stout-looking young man, of about 25, admitted on Friday, July 10th. At visit, the same day, stated that two days ago, without known cause, he had been seized with pain in the right side, cough and feverishness; he has always previously been healthy, and never subject to cough; he has, he says, within the last day or two expectorated a small quantity of blood; the expectoration is to-day scanty, frothy, mucous, with a streak or two of blood; the percussion anteriorly normal, posteriorly on the right side, dull over the acromial and upper half of the scapular regions; the axillary and upper portion of lateral region on same side are also dull to about their vertical centres; on the left side normal respiration; posteriorly over entire right side, and laterally over dull portion fine crepitating râle; elsewhere vesicular; pulse not much accelerated. (*Phosphor. 3d dilution, four times daily.*) 11th. Right side posteriorly dull over entire surface; otherwise percussion as yesterday, inferiorly and posteriorly on right side; a fine subcrepitant consonating râle over the superior portion of scapular and acromial regions, on same side; sonorous râle with occasional bronchial respiration, which is likewise heard over the right axillary and superior portion of lateral regions; left side normal; sputa rusty and scanty. 12th. Percussion posteriorly, as before, laterally clearing off; posteriorly over dull side, mucous consonating râle; crepitation laterally; sputa rusty; pulse quieter; bathed in a copious perspiration. 13th. Skin cool, and still moist, but not perspiring so much as yesterday; physical signs unchanged; sputa mucous. 14th. Percussion-sound clear anteriorly and laterally, posteriorly clearing; sputa mucous and scanty; respiration everywhere vesicular, with an occasional râle where it was formerly dull. 15th. Percussion-sound everywhere normal; an occasional crepitating râle to be heard over the right side posteriorly; respiration everywhere vesicular. 16th. The crepitating râle is to-day only audible on very full inspiration; the cough and expectoration almost entirely gone. Discharged on the 17th.

Case XVI.—J. H., a stout boy, of 10, admitted on the afternoon of Monday, July 13th. Stated next day, at visit, that he had been, previous to admission, only one day unwell. The sputa are few and rusty; cough not very troublesome; pulse full and accelerated; tongue coated, but moist; percussion posteriorly on the left side dull, right normal; over the left lateral and axillary regions tympanitic; anteriorly, on the left, slightly dull, elsewhere normal; respiration, posteriorly on the left, bronchial, elsewhere vesicular. (*Phosphor. 3d dilution, every third hour.*) 15th. His condition was unchanged. 16th. Percussion is to-day dull over the right axillary and lateral regions, otherwise unchanged; over this dull portion bronchial respiration is heard; the auscultation is otherwise unchanged; the pulse is quieter. 17th. The expectoration is to-day more catarrhal; on the left posteriorly the bronchial respiration is not so decided. 18th. Percussion on the left posteriorly still dull; laterally and anteriorly tympanitic; respiration posteriorly on the left is vesicular, preceded by a sonorous râle, elsewhere on the left laterally; rough, vesicular, puerile on the right, and anteriorly on the left normal; expectoration slight. By the 21st the vesicular respiration was audible over the entire chest, and but a slightly perceptible dullness remained on percussing the left side posteriorly, which by the 23d had, along with the cough and expectoration, entirely disappeared. On the 25th his medicine was discontinued, and on the 1st of August he was discharged.

Case XVII.—J. L., a stout boy of 16, admitted July 24th. Stated that he had been ill one day, with pain in the chest and slight cough; the expectoration is very scanty, yellow, and rusty, with a few streaks of blood, adherent; percussion anteriorly normal, posteriorly normal, except the inferior portion of right side, beneath the centre of the scapular space, where it is dull; and over this dull portion is heard on inspiration a subcrepitant râle, followed by bronchial expiration; elsewhere the respiration is normal; the pulse remains quiet; tongue moist, and skin natural. (*Phosphor. 3d dilution, every third hour.*) 25th. The râles are to-day more numerous, almost masking the bronchial expiration; the expectoration copious, of a brownish dirty-looking jelly. 27th. There is to-day a mucous râle over the left side also; the physical signs otherwise unchanged; the pulse has become somewhat accelerated. 28th. Anteriorly the percussion is dull on the right side, beneath and over the lower third of mammary region, also over the right infralateral and lower portion of the latter; a consonating râle is audible over dull space anteriorly and posteriorly; elsewhere vesicular respiration and mucous râle to be heard; skin hot; expectoration as before. 29th. Percussion-sound is anteriorly dull over entire mammary region, superiorly over right subclavicular, somewhat tympanitic; the respiration as formerly; the expectoration scarcely so copious; tongue red, inclined to be dry. 30th. The percussion improved to-day posteriorly; the râle thin, no longer consonates; the pulse is quieter; the skin moist and cool; tongue clean. 31st. Physical signs unchanged; the expectoration is scanty, colourless mucus; pulse quiet; cough not so troublesome. August 1st. Percussion posteriorly clear, anteriorly improved; respiration rough vesicular, puerile, over what was the dull posterior portion, and anterior also; expectoration mucous, still streaked with blood 3d. The percussion is to-day still slightly dull anteriorly; vesicular respiration; cough almost gone, and expectoration scanty. 4th. Discharged.

To the foregoing must be added two other cases, of which I have not full notes, viz.:

Case XVIII.—A. S., a male, aged 21, admitted May 2d. (*Phosphor. 4th dilution, four times daily.*) Discharged cured after 18 days' treatment.

Case XIX.—F. O., a male, aged 23, admitted 9th May. (*Phosphor. 3d dilution, four times daily.*) Discharged cured after 12 days' treatment.

These cases of Pneumonia give an average age of 24, an average treatment of 12·6 days, and a mortality of 15 per cent., 3 out of 19 having died.

Skoda's cases of Pneumonia, during the same time, amount to 45, his deaths to 3, giving an average of 6·6 per cent.

During this period no bloodletting of any kind was used in Dr. Skoda's wards, his treatment being, extract. graminis ℈j, or nitri puri, or sublimat. $\frac{1}{4}$ gr., by way of attempting to reduce the plasticity of the blood. He also gave occasionally pulv. Dover, gr. vj, in the course of the day. I regret that, from the patients tiring of one medicine, in no case during these three months did I see a case treated solely with extract. graminis; but hundreds could be extracted from his books treated solely with nitri puri gr. v, ad gr. xx. This seems to be his favorite remedy, though at present he is trying the new chemical theories, and gives the patients sublimate. His average mortality for the last three years, during which no bloodletting—or most rarely—has been performed, and not one leech or cupping-glass applied, is 13·7 per cent., and the recovery of the patients has always been very speedy. He told me that, in the year 1840, he treated 64 females, affected with pneumonia, with large bleedings and large doses of tartar emetic, and only lost one; yet the deaths amongst the males the same year made the total deaths amount to 1 in 8. He considers that this is about the general proportion under all treatments. The great advantage of not bleeding, he considers to be the speedy recovery. The average age was 25·3.

### OTHER DISEASES.

During the above period there were eleven cases of TUBERCULOSIS admitted. They were treated chiefly with *sulphur* 4. Two died, and two were left in the house; the others were discharged, some of them a little relieved.

Five cases of CATARRH and two of BRONCHITIS were also admitted; one of the latter following measles. They were treated chiefly with *sulphur, 4th dilution*, and, where the fever ran high, with *aconite, 3d or 4th dilution.*

Two cases of GENERAL SPASMS (at least complained of), were treated—the one with *chamomilla, 6th dilution, twice daily*, the other with *ignatia, 6th dilution, once daily*. They both recovered.

One case of CHOREA was treated with *ignatia, 3d dilution, three times daily*, and was discharged relieved.

Three cases of enlarged SCROFULOUS GLANDS were received and discharged, two in *statu quo*, one after suppuration.

One ASTHMATIC AFFECTION, without apparent cause, except hysteria, in a young girl, aged 18, was treated with *ammon. carb., 3d dilution, four times daily*, and was cured.

A case of GOITRE was treated with *iodine, 2d dilution, four times daily*, and was discharged in five days; the breathing somewhat relieved.

One case of VARIOLA MODIFICATA ran its course regularly without any medicine at all.

One case of ICTERUS, of three weeks' standing, was discharged cured after twenty days; treatment with china, 2d dilution, three times daily.

Six cases of ANGINA TONSILLARIS, slight; treated with belladonna, 3d, 4th, and 6th dilutions, three times daily, and hydrargyrum, 2d and 3d dilutions, four times daily. Average treatment not three days.

One patient with *Catarrhal Ophthalmia*, got on her entrance *sulphur, the 3d trituration, four times daily*, but the following day was not to be seen, so I suppose had been quietly discharged.

One case of *old age*, dead.

One case of *General Dropsy*, discharged after five days' treatment with *lactuca, 3d dilution, four times daily*, Improved slightly.

One case of *Exudation in the Peritoneum*, in a young boy, aged 12. The effusion was absorbed during the employment of *bryonia, the 3d dilution, four times daily*; it was followed by an inflammation and suppuration of the tunica reflexa, and the case was left under treatment.

Two cases of *Effusion into the Pleura* were discharged unimproved.

There were in all seventy-one cases in which the patients got no medicine, including several surgical cases, as ulcers, injuries, &c., and one case of burn, which was treated with a turpentine lotion. The other cases were *Chlorosis, Headaches,* slight *Stomach Complaints; Expectoration of Blood,* in one case caused by a blow, in another, by hypertrophy of the heart. . .

But I must now conclude my narrative, incomplete and imperfect as it is. I hope you will excuse the hasty manner in which it has been drawn up, from consideration of the circumstances under which I write. I was anxious to send off my letter before leaving Vienna (which I do to-morrow, August 15th), and I thought you would rather have my account, even in a rough form, early, than in a more digested form at a later period. I possess a complete list of all the patients admitted into the hospital during the three months—a statement of the medicines administered—the length of the treatment, and period of residence in the hospital, &c., which I hope to transmit to you.

I think you will see by what I have stated, that the strength of the homœopathists lies not in the greater rationality or practical superiority of their treatment, but is founded on the weakness of allopathy; that they not only do not help their patients, but—if they are strict homœopaths—are for ever shut out from helping them;—that in their treatment of acute diseases—simpler, at least, if not better than that of their opponents—their success depends entirely on the hitherto unrecognized powers of Nature — all the magic influence of their infinitesimal doses of phosphorus, &c., being emulated if not excelled by the heroic virtues of Extractum Graminis.

I remain, my dear sir, yours faithfully,

George W. Balfour.

---

### REMARKS BY THE EDITOR.

The preceding Report may be truly said to speak for itself, yet it may not be inexpedient to advert to a few of the more important particulars contained in it, especially those which bear most pointedly on the main questions to which this part of our Journal is for the present devoted.

1. In the first place, it seems clearly made out, from Dr. Balfour's account —confirmed and corroborated as it is in this point by Dr. Mühry's letter— that what is now called Homœopathy, is far from being, either in principle or practice, always or necessarily the system first promulgated by Hahnemann under this name. We think it evident, from Dr. Balfour's cases, that even Hahnemann's avowed follower, Fleischmann, sets little store by the fundamental principle, *similia similibus,* in the administration of his remedies.

2. Secondly, the facile and uniform attainment of the same triumphant results under all these varieties of the homœopathic system, points to the operation of one and the same curative agent in all—namely, our old friend, the *Vis Medicatrix.*

3. The great and manifold divisions already existing among the homœopathists, seem to lead irresistibly to the conclusion that the beginning of the end of homœopathy is already come. If we have the very best authority for believing that a house divided against itself cannot stand, we can hardly doubt that the house of Hahnemann is now tottering to its fall.

4. It is sufficiently evident from many passages in the Report that, whatever might have been the comparative general result of adopting the ordinary (allopathic) treatment in the cases related, its adoption would have been decidedly advantageous in particular instances, by affording aid to Nature in relieving distressing symptoms, which were not relieved, nor attempted to be relieved, by the homœopathic treatment. Whether homœopathy be true or false, there can be no doubt that Dr. Fleischmann's treatment was often bad.

5. But whatever may be thought of homœopathy as a doctrine, or as a general system of practice, it will not be doubted, after this Report, that the more common acute diseases may and can, during a certain period of time at least, run their course favorably enough under its administration. The general results of the treatment during the three months reported of, were, we think, such as would have satisfied many practitioners of our ordinary medicine.

6. Neither will it any longer be denied that PNEUMONIA, even in a severe form, may, under homœopathic treatment, pass through all its stages to perfect recovery.

7. The great and important practical question is—Whether or not the homœopathic remedies administered in these cases contributed in any degree —or if in some degree, in what degree—towards the cure of the diseases, particularly the cases of pneumonia? This is a question which will be answered differently by different persons. No doubt, Dr. Fleischmann and homœopathists generally will regard these cases not only as highly favorable to the claims of homœopathy, but as unquestionable proofs of its great remedial powers. We, on the contrary, in common with our reporter, see no other powers operating in these cases but the natural powers of the living system, called into action under very favorable circumstances. The general aspect of the whole cases, favorable and unfavorable alike, and the minute details of each case, convey to our mind the most perfect conviction that, throughout, Nature, not art, was the worker; and it would seem easy to convey to others the grounds of this conviction, by a minute analysis of the individual cases through their whole progress, whether the patients were getting better or getting worse, whether taking or omitting the homœopathic medicaments.

8. The same doubts as to the efficacy of the medicines employed may exist in regard to the cases of Professor Skoda. But if the advocates of homœopathy insist on the value of the evidence in favour of the decillionths of phosphorus, aconite, or bryonia, they surely have no right to reject the evidence of a precisely similar kind, in favour of Dr. Skoda's grain doses of the extractum graminis or nitre. We may perhaps be forgiven even by the most zealous partisans of ordinary medicine, particularly the abettors of the heroic system, for setting down both systems as equally effective in the case in question, that is, as having no effect at all.

9. The results of the treatment of the cases of pneumonia, both by Fleischmann and Skoda, seem to prove that bloodletting is not so essential and indispensable a remedy, even in severe cases of pneumonia, as has usually been supposed; and that its omission, in a certain proportion of cases at least, does not even retard the period of convalescence or the completion of the cure.

10. The materials supplied in Dr. Balfour's Report, like those formerly supplied in Dr. Fleischmann's, do not in any degree authorize the general conclusion that homœopathic treatment is as good as that of ordinary medicine, much less that the latter like the former is valueless, Nature being all-sufficient in the cure of diseases; but both go powerfully to corroborate the following, among other important inferences formerly deduced by us from a review of the whole question, viz.—1. That Nature is more powerful in curing diseases, and has practically a much greater share in the ordinary cure of diseases, than is commonly believed. 2. That the prevailing doctrines respecting the actions and powers of many particular medicaments and other so-called remedial agents, require reinvestigation, with a view to ascertain the truth. 3. That while this investigation is in progress, we should be cautious, even timid, in the use of medicaments and other medical means capable of exerting a powerful influence on the animal system, and therefore capable (possibly) of doing evil as well as good. 4. That in the present state of our knowledge, the Hygienic—Eclectic—Hippocratic—Natural system of treating diseases, is the only one that can be justified or safely followed.

